# Second-Line Bismuth-Containing Quadruple Therapy for *Helicobacter* *pylori* Infection: A 12-Year Study of Annual Eradication Rates

**DOI:** 10.3390/jcm10153273

**Published:** 2021-07-24

**Authors:** Kiwon Shin, Min-Jae Cho, Jung-Hwan Oh, Chul-Hyun Lim

**Affiliations:** Department of Internal Medicine, College of Medicine, The Catholic University of Korea, Seoul 06591, Korea; mrsunset@empal.com (K.S.); heehee2210@hanmail.net (M.-J.C.); ojh@catholic.ac.kr (J.-H.O.)

**Keywords:** bismuth, breath test, duration of therapy, eradication, *Helicobacter pylori*

## Abstract

Background: Bismuth-containing quadruple therapy (BQT) consisting of a proton-pump inhibitor (PPI), bismuth, metronidazole and tetracycline is recommended as a second-line treatment for *Helicobacter pylori* (*H. pylori*) infection when PPI-based standard triple therapy (STT) consisting of a PPI, amoxicillin and clarithromycin is unsuccessful. The purpose of this study was to analyze the long-term results of BQT as a second-line therapy to determine its effectiveness. Methods: This study included 643 subjects who failed first-line STT and received 7 or 10–14 days of BQT as a second-line therapy. We retrospectively analyzed the annual *H. pylori* eradication rates, demographic factors and adverse events. Results: The overall eradication rates by intention-to-treat (ITT) and per-protocol (PP) analyses were 80.7% (519/643) and 93.3% (519/556), respectively. By PP analysis, the eradication rates for 2008–2011, 2012–2015, and 2016–2019 were 93.3%, 91.0%, and 96.4%, respectively (*p* = 0.145). There were no significant differences between the 7-day group and the 10–14-day group in both the ITT (79.7% vs. 86.0%, *p* = 0.148) and the PP analyses (92.7% vs. 96.6%, *p* = 0.187). A multivariate analysis showed that current smoking was associated with eradication failure. Eighty-nine subjects (16.0%) suffered adverse events, mainly gastrointestinal symptoms, but only six cases were severe. Conclusions: BQT as a second-line therapy is an effective treatment for *H. pylori*. Treatment for 10–14 days showed a higher eradication rate compared with a 7-day regimen, but not significantly.

## 1. Introduction

*Helicobacter pylori* (*H. pylori*) infection is known to be associated with diverse gastrointestinal diseases including chronic gastritis, peptic ulcers, gastric mucosa-associated lymphoid tissue and gastric cancer [1,2]. Furthermore, *H. pylori* infection has also been reported to be linked to several extra-gastric diseases, including iron deficiency anemia, primary immune thrombocytopenia and vitamin B12 deficiency anemia [3]. The eradication of *H. pylori* has been reported to be beneficial not only for the treatment of peptic ulcers, but also for the treatment and prevention of *H. pylori*-associated diseases such as gastric cancer [4]. Many current guidelines recommend *H. pylori* eradication for these reasons, and it is considered to be especially important in areas of high gastric cancer prevalence [5,6,7].

Various combination therapies are recommended for *H. pylori* eradication. Generally, proton pump inhibitor (PPI)-based standard triple therapy (STT) of PPI, amoxicillin and clarithromycin has been commonly recommended as the first-choice treatment. If it fails, bismuth-containing quadruple therapy (BQT) consisting of PPI, bismuth, metronidazole and tetracycline is recommended as a second-line therapy [5,6,7,8,9]. In Korea, recent reported data of *H. pylori* resistance rates against clarithromycin, metronidazole, amoxicillin, tetracycline and levofloxacin were 17.8~45.9%, 29.5~43.2%, 8.1~9.5%, 0~16.2% and 37.0~62.2%, respectively [10,11], and there has been a decrease in efficacy of first-line STT due to an increased resistance to clarithromycin [12]. Although the need for BQT as a second-line therapy has increased, there is controversy about its efficacy due to a high resistance to metronidazole in Korea [10]. The metronidazole resistance rate reported in Korea is higher than in the US and Europe [13,14]. Furthermore, recent studies in Korea have reported suboptimal results or a decreasing trend of annual eradication rates of *H. pylori* with BQT [15,16,17].

The aim of the present study was to confirm the long-term effectiveness of BQT as a second-line therapy for *H. pylori*. In addition, we investigated risk factors related to the failure of second-line therapy.

## 2. Materials and Methods

### 2.1. Subjects

We retrospectively reviewed the medical records of our center from January 2008 to December 2019. We enrolled patients diagnosed with an *H. pylori* infection who had received second-line BQT due to the failure of first-line STT. *H. pylori* positivity was assessed using the ^13^C-urea breath test (UBT) (UBiTkit; Otsuka Pharmaceutical, Tokyo, Japan) and histopathologic examination findings before and after the eradication therapy. Compliance was assessed by counting the number of remaining pills via direct questioning or a questionnaire form. Poor compliance was defined as taking 80% of the prescribed medicine. Loss to follow-up was defined as unknown results regarding the eradication success or failure. We also investigated the following demographic features: age, sex, smoking, alcohol, type 2 diabetes mellitus, hypertension and endoscopic findings. This study was approved by the IRB of Eunpyeong St. Mary’s Hospital, Seoul, Korea (PC20RISI0218).

### 2.2. BQT

All subjects received 7, 10, or 14 days of treatment with BQT consisting of standard-dose PPI twice daily, metronidazole 500 mg three times daily, bismuth 120 mg and tetracycline 500 mg four times daily. *H. pylori* eradication was confirmed by UBT at least 4 weeks after the completion of treatment. No PPI, H2 blockers, or bismuth were allowed within 4 weeks of the UBT. Adverse reactions to the eradication therapy were investigated based on medical records. Severe adverse events were defined as those requiring the discontinuation of treatment.

### 2.3. Statistical Analysis

The *H. pylori* eradication rate was analyzed by intention-to-treat (ITT) and per-protocol (PP) analyses. The trends in annual eradication rates were analyzed using the Cochran–Armitage trend test. Categorical variables were analyzed using the chi-squared test, and continuous variables were analyzed using the independent t-test. Univariate and multivariate logistic regression tests were used for the analysis of risk factors, which were expressed as an odds ratio (OR) and 95% confidence interval (CI). Statistical significance was defined as a *p*-value < 0.05. All statistical analyses were performed using SAS v. 9.4 for Microsoft Windows (SAS Institute Inc., Cary, NC, USA).

## 3. Results

### 3.1. Characteristics

**Figure 1:**
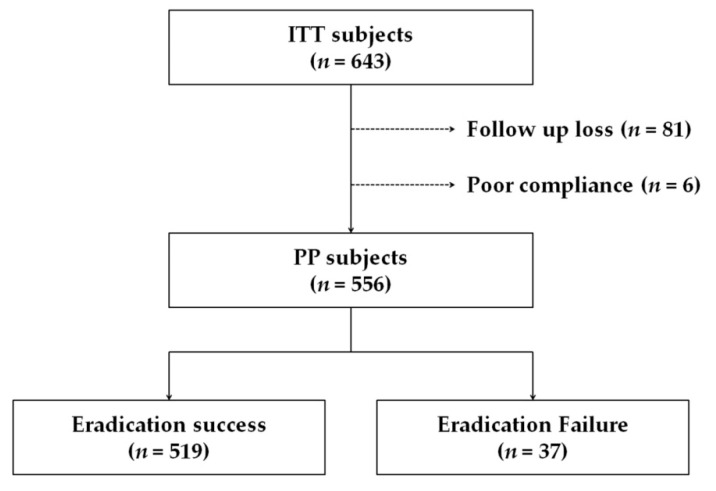
Flowchart of the study. ITT, intention-to-treat; PP, per-protocol.

This study included an ITT population of 643 subjects. Of these, 81 were lost to follow-up and six were excluded because of poor compliance (Figure 1). The study included 265 males (41.2%); the mean age was 57.1 ± 12.3 years (18–86 years). Endoscopic findings at the time of the eradication therapy initiation were gastritis (54.6%), peptic ulcer (42.9%), stomach neoplasm such as early gastric cancer, and gastric adenoma (2.5%). Among the cohort, 139 subjects (21.6%) had hypertension and 80 subjects (12.4%) had type 2 diabetes mellitus (Table 1).

### 3.2. Eradication Rates

#### 3.2.1. Eradication Rates by Year

**Figure 2:**
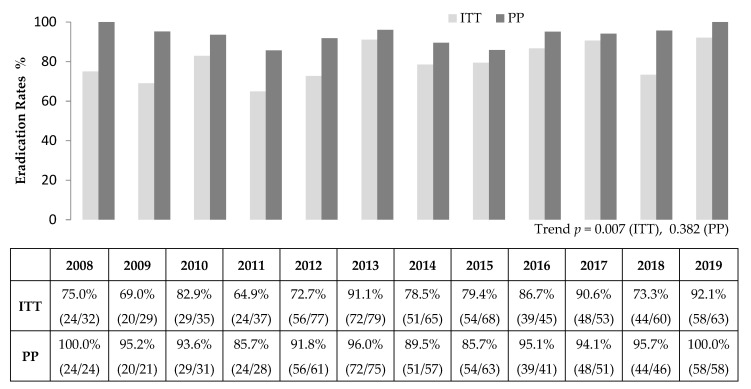
Annual *Helicobacter pylori* eradication rates of second-line bismuth containing quadruple therapy. ITT, intention-to-treat; PP, per-protocol.

The overall eradication rates by ITT and PP analyses were 80.7% (519/643) and 93.3% (519/556), respectively. The annual eradication rates for 2008–2019 ranged from 64.9% to 92.1% in the ITT analysis and from 85.7% to 100% in the PP analysis (Figure 2). In the ITT analysis, the eradication rate increased over time (*p* = 0.004), whereas there was no statistically significant difference over time in the PP analysis (*p* = 0.146) (Table 2).

#### 3.2.2. Eradication Rates by Treatment Duration

**Figure 3:**
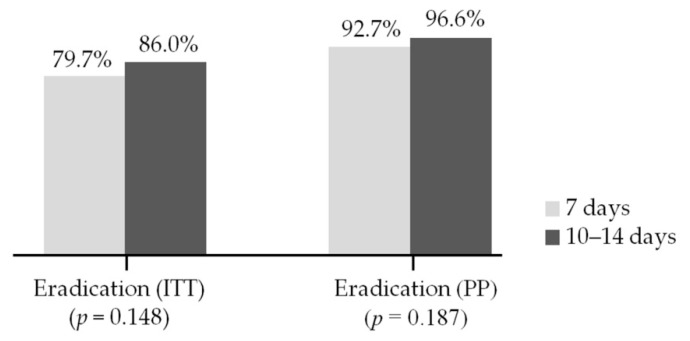
Comparison of eradication rate between the 7-day and the 10–14-day group. ITT, intention-to-treat; PP, per-protocol.

Eradication rates in the 7-day group were 79.7% (433/543) in the ITT analysis and 92.7% (433/467) in the PP analysis. Eradication rates in the 10–14-day group were 86.0% (86/100) in the ITT analysis and 96.6% (86/89) in the PP analysis. There were no significant differences between the 7-day and the 10–14-day group in both the ITT (*p* = 0.148; 95% CI, 0.85–2.85) and PP analyses (*p* = 0.187, 95% CI, 0.68–7.49) (Figure 3).

### 3.3. Factors Related to Eradication Failure

Univariate and multivariate analyses showed that current smoking was associated with eradication failure (OR, 2.92; 95% CI, 1.36–6.24; *p* = 0.006). There was no significant relationship between eradication failure and other factors including age, sex, alcohol, hypertension and diabetes (Table 3).

### 3.4. Adverse Events

Among the 562 subjects who completed follow-up, 95 patients (16.9%) complained of events after BQT. Severe adverse events requiring the discontinuation of treatment occurred in six patients. The most common reported adverse event was nausea or vomiting (5.9%), followed by diarrhea or loose stool (3.6%), abdominal pain or discomfort (3.0%), and headache or dizziness (2.0%) (Table 4). There was no statistical significance between the 7-day and the 10–14-day groups in the PP analysis (15.0% vs. 21.4%, *p* = 0.134).

## 4. Discussion

The overall eradication rate of second-line BQT was 80.7% by the ITT analysis and 93.3% by the PP analysis. This result is important because it meets the suggested successful eradication rate [7,18] despite the high antibiotic resistance in Korea [19]. Our data also suggest that annual eradication rates in the PP analysis did not decrease over time despite the trend of increasing antibiotic resistance in our geographic area. This finding is consistent with a recent study in Korea that reported no decrease in eradication rates of second-line BQT from 2003 to 2018 [20], although that study analyzed data from a 14-day regimen. Another study from 2006 to 2015 reported a similar result [21], but the number of subjects was relatively small (231 subjects). Interestingly, although there is a difference in the rate of antibiotic resistance in various geographic areas, BQT shows successful results and is recommended as a first- or second-line therapy in most areas [5,6,7,8,9,22]. Recent European data (Hp-EuReg) also showed similar results to this study in second-line BQT [23,24].

We found that the eradication rates determined by ITT have been relatively high in recent years, which might be due to our recent efforts to maintain patient follow-up. Moreover, we have tried to maintain medication compliance by proactively explaining possible adverse events during treatment. At the same time, we emphasize the importance of the UBT to confirm eradication of *H. pylori* after the completion of treatment, which may make patients continue with follow-up. Taken together, we suggest that these strategies might be important factors that mediate the successful eradication of *H. pylori*.

There has been debate about the duration of second-line BQT. The 2013 Korean guideline recommends a duration of approximately 7 to 14 days for second-line BQT [7]. The more recently published 2020 Korean guideline recommends BQT for 14 days as a second-line therapy [8], but this recommendation has not yet been widely accepted in clinical settings. Considering the potential for adverse events and difficulties with compliance, some clinicians prefer a 7-day regimen. On the other hand, others prefer a 10–14-day regimen because a longer duration might overcome metronidazole resistance [25]. Only a few small-sized prospective studies in Korea have compared eradication rates by treatment duration, and each reported different results. In two studies, the eradication success rate of the 14-day treatment was statistically higher than the 7-day treatment [26,27], whereas another study reported that 7-day therapy was as effective as 14-day therapy [28]. A more recent prospective study reported that 14 days of therapy tended to show a higher eradication rate compared with 7 days of therapy in both ITT (79.6% vs. 90.4%) and PP analyses (91.7% vs. 100%), but there were no significant differences [29]. Similarly, in the present study, the eradication success rate in the 10–14-day group was higher than it was in the 7-day group, although there was no statistical significance. Although these data suggest that a longer duration of treatment may be recommended for patients with good compliance and no major adverse events, there is a need for further larger prospective studies that focus on eradication success rates and adverse event rates by treatment duration in order to ascertain which treatment duration is better.

Some factors such as age, sex, smoking status and type 2 diabetes mellitus have been reported to be associated with the eradication rate [30,31,32]. Our study showed that only smoking was positively associated with eradication failure. This result is consistent with earlier studies including a meta-analysis of 22 studies of 5538 patients, and a recent large-cohort-size study of 58,130 patients [31,33]. Some possible mechanisms have been suggested to explain the effects of smoking on eradication failure. First, smoking is known to decrease gastric blood flow [34], which might reduce the delivery of antibiotics to the gastric mucosa and consequently lead to treatment failure. Second, smoking stimulates acid secretion [35], which might increase the proportion of nonreplicative bacteria that could reduce the effectiveness of antibiotics. Third, smoking might modulate the activity of specific CYP450 isoenzymes involved in the metabolism of PPI [36]. Lastly, smoking may simply be a marker of poor compliance [37]. In previous studies and in this study, it was not clear whether current smokers actually smoked during treatment. It would be useful to clarify whether temporary smoking cessation during medication makes a difference to eradication rates. Other factors including age, sex, alcohol, hypertension, and type 2 diabetes mellitus did not appear to be associated with treatment failure in this study.

Some therapy-induced adverse events were observed. Most of them were related to gastrointestinal symptoms including nausea, dyspepsia, bloating and diarrhea. Some nongastrointestinal symptoms such as headache and dizziness were also reported, but no severe neurologic symptoms resembling bismuth-related encephalopathy were observed. The symptoms were mostly well tolerated, and no specific treatments were needed. Previous studies have also reported that BQT is safe and well tolerated [17,38].

There are some limitations of this study. It was performed at a single center and a non-negligible number of patients were lost to follow-up, which made a relevant difference in the eradication rate between ITT and PP. In addition, because it was a retrospective study, there were considerable missing data relating to factors such as compliance, smoking and drinking status, underlying disease, and adverse events. There were also differences in the kinds of PPI and diagnostic methods. All of these subtleties might affect the results. Furthermore, antibiotic resistance is thought to be an important factor influencing the eradication rate [39]. Here, appropriate analysis was not possible because *H. pylori* culture and antibiotic susceptibility tests were not conducted in our center.

A strength of this study is that we measured annual eradication rates over a long period of time in a relatively large number of study subjects who had failed first-line STT and received second-line BQT. These data represent the real-world clinical situation in which the recently emerging clarithromycin resistance test cannot be performed. A recent meta-analysis of 26 studies from Korea found that pooled eradication rates of first-line STT were 71.6% in ITT analysis and 79.6% in PP analysis [8]. Applying our data here, the expected eradication success rates are 86.5% in ITT analysis and 98.7% in PP analysis, assuming that *H. pylori*-positive individuals receive first-line STT and second-line BQT if necessary. This suggests that the widely used process of prescribing second-line BQT in the case of first-line STT failure remains effective in Korea. In addition, we suggest that the effort to explain in detail to patients the importance of taking the drugs as prescribed, the possible adverse events during treatment, and the need for a confirmation test after treatment could improve the eradication rate. A more active and standardized effort is thought to be able to improve compliance, which is considered the weak point of BQT.

## 5. Conclusions

BQT as a second-line therapy remains an effective therapy in Korea. Current smoking was found to be associated with eradication failure. A period of 10–14 days of therapy showed a higher eradication rate compared with 7 days, although there was no statistical significance. Further larger-scale multicenter prospective studies are needed in the future.

## Figures and Tables

**Table 1:** Baseline characteristics of patients.

	ITT	PP
Number	643	556
Age (y) mean ± SD	57.1 ± 12.3	57.4 ± 12.2
Sex (%)		
Male	265 (41.2)	220 (39.6)
Female	378 (57.8)	336 (60.4)
Diabetes mellitus (%)	80 (12.4)	62 (11.2)
Hypertension (%)	139 (21.6)	119 (21.4)
Endoscopic findings (%)		
Peptic ulcer	351 (54.6)	299 (53.8)
Gastritis	276 (42.9)	243 (43.7)
EGCa + gastric adenoma	16 (2.5)	14 (2.5)
Cigarette smoking (%)	89/606 (14.7)	70/525 (13.3)
Alcohol intake (%)	169/606 (27.9)	142/525 (27.0)

ITT, intention-to-treat; PP, per-protocol; SD, standard deviation; EGCa, early gastric cancer.

**Table 2:** Cumulative eradication rates per year-periods.

Year	Intention-to-Treat Eradicated/Total (%)	Per-Protocol Eradicated/Total (%)
2008~2011	97/133 (72.9)	97/104 (93.3)
2012~2015	233/289 (80.6)	233/256 (91.0)
2016~2019	189/221 (85.5)	189/196 (96.4)
Total	519/643 (80.7)	519/556 (93.3)

**Table 3:** Logistic regression analysis to predict factors for eradication rate. (Per-Protocol).

	Univariate Analysis			Multivariate Analysis		
	OR	95% CI	*p*-Value	OR	95% CI	*p*-Value
Age (years)	1.01	0.98, 1.04	0.620			
Male (ref. Female)	0.82	0.41, 1.64	0.569			
Smoking (ref. never-smoker)	2.92	1.36, 6.24	0.006	2.92	1.36, 6.24	0.006
Alcohol (ref. non-alcohol)	0.90	0.41, 1.97	0.793			
Type 2 Diabetes mellitus (ref. non-T2DM)	0.96	0.33, 2.82	0.946			
Hypertension (ref. non-hypertension)	1.39	0.66, 2.97	0.389			
10–14 days (ref. 7 days)	0.44	0.13, 1.48	0.186			

OR, odds ratio; CI, confidential interval; DM, diabetes mellitus; HTN, hypertension.

**Table 4:** Adverse events during quadruple therapy.

	Patients (%), *n* = 562
Nausea, vomiting	33 (5.9%)
Loose stool, diarrhea	20 (3.6%)
Abdominal discomfort or pain	14 (3.0%)
Dyspepsia, poor oral intake	12 (2.1%)
Headache, dizziness	11 (2.0%)
Dry mouth	6 (1.1%)
General weakness	6 (1.1%)
Others	10 (1.8%)

## Data Availability

The datasets generated and/or analyzed during the current study are not publicly available due to our IRB policy but are available from the corresponding author upon reasonable request.
